# Mechanism and consequences of herpes simplex virus 1-mediated regulation of host mRNA alternative polyadenylation

**DOI:** 10.1371/journal.pgen.1009263

**Published:** 2021-03-08

**Authors:** Xiuye Wang, Liang Liu, Adam W. Whisnant, Thomas Hennig, Lara Djakovic, Nabila Haque, Cindy Bach, Rozanne M. Sandri-Goldin, Florian Erhard, Caroline C. Friedel, Lars Dölken, Yongsheng Shi

**Affiliations:** 1 Department of Microbiology and Molecular Genetics, School of Medicine, University of California, Irvine, California, United States America; 2 Institute for Virology and Immunobiology, Julius-Maximilians-University Würzburg, Germany; 3 Institute of Informatics, Ludwig-Maximilians-Universität München, Germany; 4 Helmholtz Institute for RNA-based Infection Research, Würzburg, Germany; Southwestern Medical Center, UNITED STATES

## Abstract

Eukaryotic gene expression is extensively regulated by cellular stress and pathogen infections. We have previously shown that herpes simplex virus 1 (HSV-1) and several cellular stresses cause widespread disruption of transcription termination (DoTT) of RNA polymerase II (RNAPII) in host genes and that the viral immediate early factor ICP27 plays an important role in HSV-1-induced DoTT. Here, we show that HSV-1 infection also leads to widespread changes in alternative polyadenylation (APA) of host mRNAs. In the majority of cases, polyadenylation shifts to upstream poly(A) sites (PAS), including many intronic PAS. Mechanistically, ICP27 contributes to HSV-1-mediated APA regulation. HSV-1- and ICP27-induced activation of intronic PAS is sequence-dependent and does not involve general inhibition of U1 snRNP. HSV1-induced intronic polyadenylation is accompanied by early termination of RNAPII. HSV-1-induced mRNAs polyadenylated at intronic PAS (IPA) are exported into the cytoplasm while APA isoforms with extended 3’ UTRs are sequestered in the nuclei, both preventing the expression of the full-length gene products. Finally we provide evidence that HSV-induced IPA isoforms are translated. Together with other recent studies, our results suggest that viral infection and cellular stresses induce a multi-faceted host response that includes DoTT and changes in APA profiles.

## Author summary

Viral infections profoundly alter host cell gene expression. It is important to understand both how viruses hijack the host cell machineries to express their own genes and how host cells respond to viral infection for defense. We have previously shown that herpes simplex virus-1 (HSV-1) blocks host cell transcription termination, at least in part, through the viral immediate early protein ICP27. Here we show that HSV-1 infection also alters mRNA 3’ end formation and promotes the formation of truncated mRNAs. Some of these aberrant mRNAs are exported into the cytoplasm and translated. This viral activity requires ICP27 and other viral factors. Our study, together with other recent reports, suggests that viral infections and cellular stress elicit similar responses in mammalian cells.

## Introduction

The 3’ ends of the vast majority of eukaryotic mRNAs are formed through cleavage and polyadenylation [1–3]. In mammals, poly(A) sites (PAS) are defined by several cis-elements, including the AAUAAA hexamer, the U/GU-rich downstream element, and other auxiliary sequences. These sequences recruit RNA 3’ processing factors CPSF, CstF, CFIm, CFIIm, and the poly(A) polymerase to form the 3’ processing complex. RNA 3’ processing occurs co-transcriptionally and it plays an essential role not only in RNA biogenesis, but also in transcription termination by RNA polymerase II (RNAPII) [4–6]. According to the “allosteric model” of transcription termination, the transcription machinery undergoes a transformation upon passing through a PAS, which primes RNAPII for termination. Alternatively, the “torpedo model” posits that the unprotected 5’ end of RNA generated by the 3’ processing cleavage step is recognized by the exoribonuclease Xrn2. Xrn2-mediated degradation of the nascent RNA ultimately leads to transcription termination. Thus, in both models, RNA 3’ processing plays a central role in transcription termination.

RNA 3’ processing also plays an important role in gene regulation. The transcripts of over 70% of human genes can be cleaved and polyadenylated at multiple alternative PAS, a process called alternative polyadenylation (APA) [7–9]. Different APA isoforms from the same gene may encode distinct proteins and/or contain different 3’ untranslated regions (UTRs). 3’ UTRs are hot spots for regulation: they harbor target sites for microRNAs, binding sites for RNA-binding proteins, RNA localization signals, and they can function as protein assembly platforms. Thus, APA isoforms from the same gene could be differentially regulated. Recent studies have provided evidence that APA plays important roles in a wide variety of biological processes and aberrant APA regulation has been linked to a number of diseases, including cancer and neurological disorders [10]. Many APA regulators have been identified, including the core RNA 3’ processing factors, splicing factors, and RNA-binding proteins [11]. For example, U1 snRNP has been shown to inhibit premature cleavage/polyadenylation at intronic PAS, thereby protecting transcript integrity globally [12]. Despite recent progress, however, the regulatory mechanisms and functional consequences of APA remain poorly understood.

Both RNA 3’ processing and transcription termination are highly regulated. For example, we have previously shown that HSV-1 infection leads to a widespread disruption of transcription termination (DoTT) [13,14]. Influenza virus (IAV) was reported to elicit a similar response [15]. The Steitz lab observed a transcription termination defect in cells exposed to salt/osmotic stress that leads to the production of transcripts downstream of genes (DoGs) [16]. A comparative analysis showed that virus-induced DoTT and stress-induced DoGs are highly related [17]. Although the mechanism for DoTT/DoGs remains unclear, we have recently shown that the viral immediate early factor ICP27 contributes to HSV-1-induced DoTT by directly binding to the RNA 3’ processing factor CPSF and inhibiting the cleavage step [13]. Meanwhile, several groups reported that virus infections, such as the human cytomegalovirus (HCMV) and vesicular stomatitis virus (VSV), or stress can induce global APA changes [18–20]. However, the relationship between virus- or stress-induced APA and DoTT/DoG remains unclear. In this study, we integrated time-resolved global APA profiling, nascent RNA sequencing, cell fractionation and RNA sequencing, and ribosome profiling (Ribo-seq) data in HSV-1-infected cells to elucidate the scope, mechanism, and functional impact of virus-induced APA changes and DoTT.

## Results

### HSV-1 infection induces widespread and dynamic APA changes

**Fig 1:**
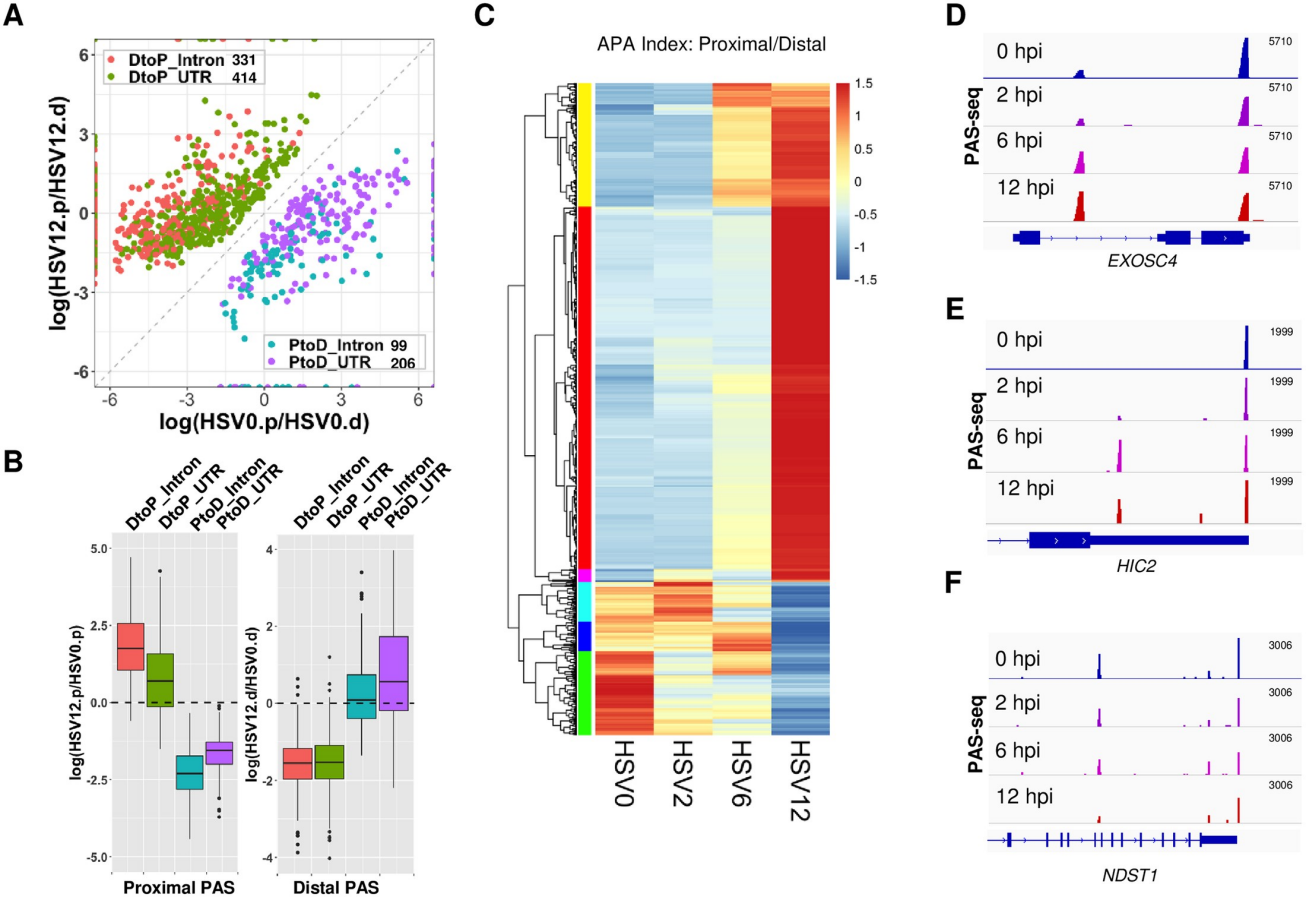
HSV-1 infection induces widespread and dynamic APA changes. (A) A scatter plot of HSV-1-induced significant APA changes in HeLa cells (FDR < 0.05, at least 15% of the transcripts shifted). HSV0.p or HSV12.p: read counts for the proximal PAS in cells at 0 or 12 hpi; HSV0.d or HSV12.d: read counts for the distal PAS in cells at 0 or 12 hours post infection. DtoP: a distal to proximal shift; PtoD: a proximal to distal shift. Intron: shifts involving an intronic PAS. UTR: both PAS are located in the 3’ UTR. (B) Relative read count changes at proximal (.p) or distal (.d) PAS in the four groups of APA shifts. (C) A heat map showing the APA index (proximal/distal read count ratio) of all the genes with significant APA shifts as shown in (A). Data was scaled by row. Color bars on the left denote the 6 groups that displayed similar kinetic patterns. (D-F) PAS-seq tracks of example genes.

To determine if and how the global APA profile of host genes is altered during HSV-1 infection, we performed PAS-seq analysis of HeLa cells at 0, 2, 6, and 12 hours post-infection (hpi). PAS-seq is a method developed in our laboratory for quantitatively mapping RNA poly(A) junctions [21]. Briefly, poly(A)+ RNAs are fragmented to ~200 nucleotide (nt) fragments and reverse transcribed using oligo(dT) primers, and then the poly(A) junction-containing DNAs are amplified for high throughput sequencing. This method has been used extensively for profiling global APA [22,23]. By comparing the APA profiles of cells at 0 and 12 hpi, we detected significant APA changes in 1,050 genes (FDR < 0.05, impacting at least 15% of transcripts, see Methods for details). In 745 genes (71%), polyadenylation shifted to proximal PAS (Distal-to-Proximal or DtoP) and 305 (29%) showed changes in the opposite direction (Proximal-to-Distal or PtoD) (Fig 1A). HSV-1 infection led to the apparent activation of many PAS that are unused in uninfected cells. For example, among the DtoP changes, 188 genes (25%) shifted to a proximal PAS that was not used in uninfected cells. 44 genes (14%) of those displayed PtoD shifts activated a previously unused distal PAS. Additionally, a significant portion of HSV-1-induced changes involved intronic PAS. For example, among the DtoP changes, 331 (44%) shifted to a proximal intronic PAS (DtoP_Intron) while 32% of PtoD genes shifted from a proximal intronic PAS to a PAS in the 3’ UTR (PtoD_Intron, Fig 1A). APA profile changes could be due to differential PAS selection during transcription, differential degradation of APA isoforms, a selective loss of proximal or distal PAS due to read-through transcription, or a combination of all factors. To begin to understand the cause of the observed APA changes in HSV-1-infected cells, we compared the PAS-seq reads (normalized by sequencing depths of the host transcriptomes) at proximal and distal PAS of genes that displayed significant APA changes. As shown in Fig 1B, DtoP changes were accompanied by a relative increase in proximal PAS reads and a relative decrease in distal PAS reads. Conversely, PtoD changes involving only UTR PAS were accompanied by the opposite changes. In addition to ratios, DtoP changes were accompanied by a net increase in the normalized read counts at proximal PAS and a net decrease at the distal PAS, while the opposite changes were observed for PtoD shifts (S1A Fig). These results provided evidence that the HSV-1-induced APA changes are not caused solely by preferential degradation or loss of specific APA isoforms, but may require a shift in PAS usage. The underlying mechanism will be further addressed below.

To determine the kinetics of APA changes during HSV-1 infection, we compared the APA index (read count ratio between proximal and distal PAS) of the 1,050 genes. The greatest shifts in APA profile occurred between 6 and 12 hpi (Fig 1C and S1B Fig). However, multiple different kinetic patterns were observed for the timing and magnitude of APA changes (Fig 1C, see the colored sidebars for classification), indicating that multiple mechanisms are involved in regulating the APA of host genes. Three examples were provided to illustrate the different kinetic groups. For example, polyadenylation of the *EXOSC4* transcripts shifted from a PAS in the 3’ UTR to a proximal intronic PAS (Fig 1D). The majority of the APA shift occurred between 2 and 6 hpi and a modest further shift was observed between 6 and 12 hpi. Similarly, polyadenylation of *HIC2* transcripts shifted to a proximal intronic PAS between 2 and 6 hpi. However, the usage of this intronic PAS decreased subsequently (Fig 1E). Finally, a PtoD shift was observed for *NDST1* transcripts and the majority of the APA change occurred between 6 and 12 hpi (Fig 1F). Together, these data demonstrated that HSV-1 infection induces widespread APA changes, the majority of which shift from distal to proximal PAS. These APA changes follow multiple kinetic patterns, indicating that different mechanisms might be involved in HSV-1-mediated APA regulation of host genes.

### The relationship between the HSV-1-induced APA changes and transcription

**Fig 2:**
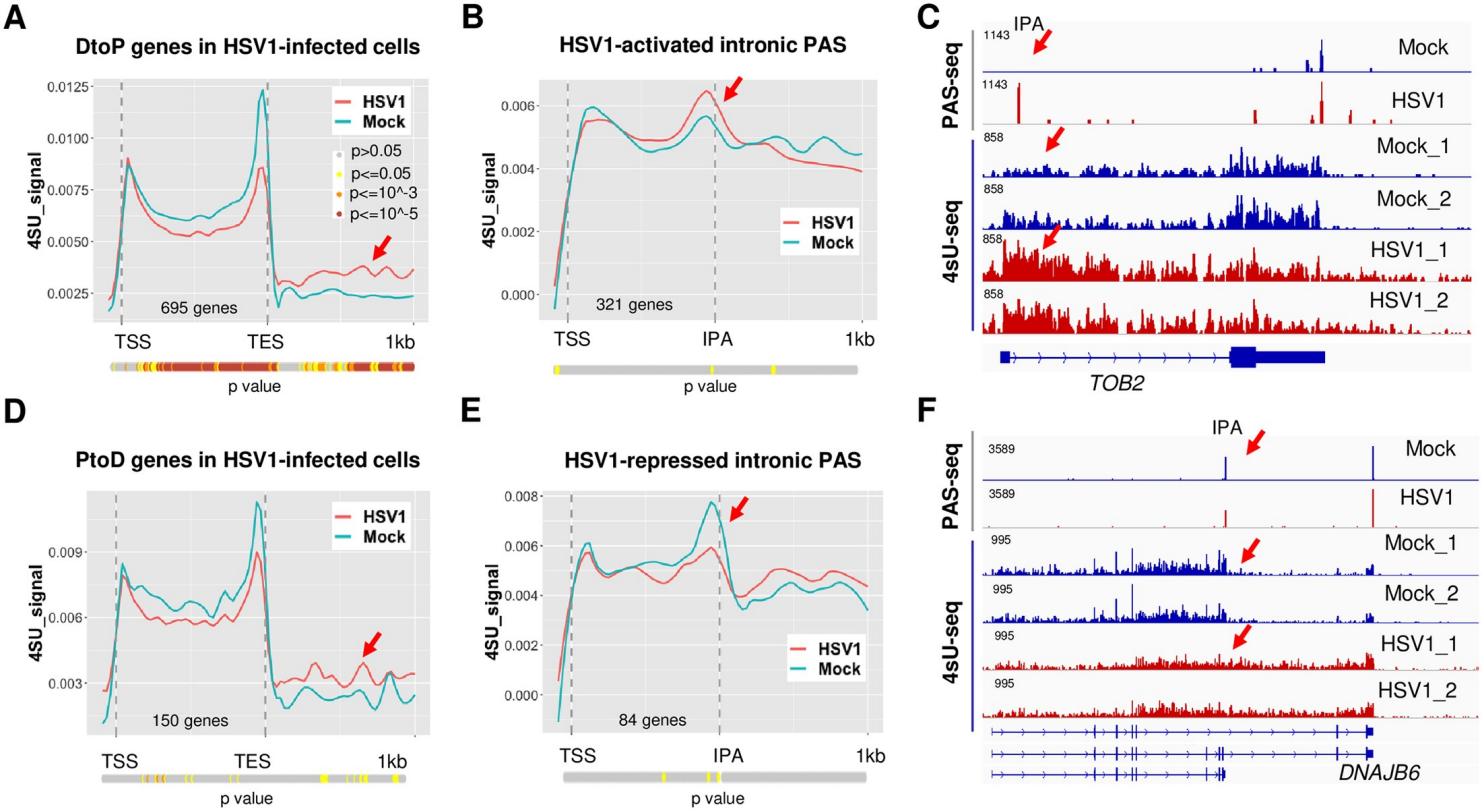
HSV-1-induced APA changes occur, at least in part, co-transcriptionally. (A) 4sU-seq signals of genes that displayed DtoP APA shifts in HSV-1-infected HeLa cells (8 hpi). TSS: transcription start site. TES: transcription end site. P values for the difference between the 4sU-seq signals at each position in mock and HSV1-infected cells were calculated using Wilcoxon rank sum test and plotted as a color-coded bar below the plot. The color scheme is shown in the inset. (B) 4sU-seq signals at genes that displayed DtoP APA shifts in HSV-1 infected cells involving intronic PAS. IPA: intronic polyadenylation site. (C) PAS-seq and 4sU-seq tracks of *TOB2*. Red arrows point to the IPA region. (D) 4sU-seq signals at genes that displayed PtoD APA shifts in HSV-1 infected cells. (E) 4sU-seq signals at genes that displayed PtoD APA shifts in HSV-1 infected cells involving intronic PAS. (F) PAS-seq and 4sU-seq tracks of *DNAJB6*.

HSV-1-induced APA changes could be due to changes in PAS selection during transcription and/or selective loss of individual APA isoforms. To distinguish between these mechanistic models, we directly compared our PAS-seq data with nascent RNA sequencing (4sU-seq) data, which provides information on transcription activities [13]. We focused our analyses on the gene body as well as 1 kilobase (kb) downstream of the transcript end site (TES) in order to monitor both transcription elongation and termination. To avoid detecting signals from neighboring genes, we selected the APA genes that do not overlap with other genes within the 1 kb downstream region (508 DtoP and 130 PtoD genes). Meta-analyses of 4sU-seq signals in mock or HSV-1-infected cells along the genes that showed HSV-1-induced higher usage of upstream PAS (DtoP) revealed two interesting differences. First, although the 4sU-seq signals were similar at transcription start sites (TSS), the signal intensities were significantly lower within the gene body in HSV-1-infected cells (Fig 2A, p values for each position were calculated using Wilcoxon rank sum method and shown as a color-coded bar below the plot), indicating loss of transcription activity within this region. Second, the 4sU-seq signals downstream of transcription end site (TES) in HSV-1-infected cells were higher than those in mock treated cells, consistent with DoTT (Fig 2A, marked by a red arrow). To better monitor potential changes in transcription activities near the regulated PAS, we focused on DtoP shifts involving proximal IPA that are used in at least 20% of the transcripts. Importantly, accumulation of 4sU-seq signals were observed at these IPA (p < 0.05, Wilcoxon rank sum test), followed by a decrease in HSV-1-infected cells (Fig 2B, a quantitative comparison for individual PAS is shown in S2 Fig). This pattern is a hallmark of transcriptional termination [5], suggesting that the observed higher PAS-seq signals at these IPA are, at least in part, due to higher usage of these PAS during transcription. This is exemplified by the gene *TOB2* (Fig 2C). Here, an IPA in *TOB2* was activated in the HSV-1-infected cells. Concomitantly, 4sU-seq signals accumulated in this region followed by a decrease in HSV-1-infected cells, consistent with transcriptional termination. Significantly higher 4sU-seq signals were also observed downstream of the TES consistent with impaired PAS usage and read-through transcription at the canonical downstream PAS (Fig 2C). As a comparison, we also plotted the 4sU-seq signals in mock and HSV-1-infected cells for genes that displayed PtoD APA changes. Different from the DtoP genes (Fig 2A and 2B), the 4sU-seq signals from mock and HSV-1-infected cells were similar over the gene body for PtoD genes (Fig 2D), indicating that there was no early transcription termination. Additionally, the transcription termination defect was less significant (Fig 2D, marked by a red arrow). When examining the 4sU-seq signals at the IPA of PtoD genes, we observed the opposite pattern than the DtoP genes. A peak followed by a valley pattern was detected at these IPA in mock treated cells (Fig 2E, blue line), indicating polyadenylation at these sites was accompanied by transcriptional termination. By contrast, 4sU-seq signals in HSV-1-infected cells were relatively flat at these intronic PAS, indicating transcriptional readthrough (Fig 2E, red line). This pattern is exemplified by the *DNAJB6* gene (Fig 2F). A major decrease in 4sU-seq signals were observed at the IPA of DNAJB6 in mock-treated cells, consistent with transcription termination at this site (Fig 2F). In HSV-1 infected cells, transcription extended beyond the IPA, accompanied by a shift in polyadenylation to the downstream PAS (Fig 2F). Together, these data suggest that the HSV-1-induced changes in APA profiles are, at least in part, caused by changes in PAS usage during transcription.

### ICP27-dependent and -independent APA changes during HSV-1 infection

**Fig 3:**
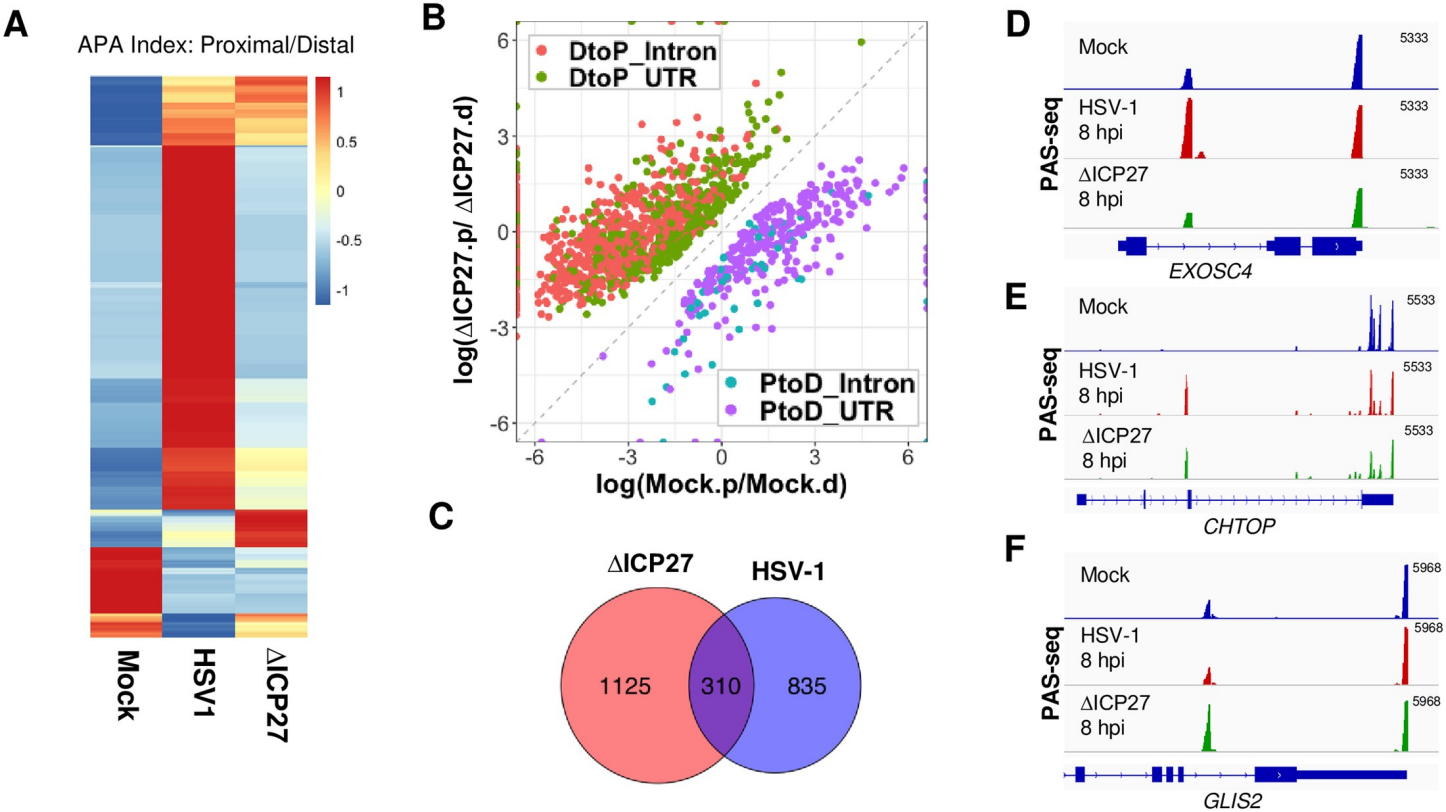
ICP27-dependent and -independent HSV-1-induced APA changes. (A) A heat map showing the APA index of all the genes that displayed significant APA changes as shown in Fig 1A in mock, wild-type or ΔICP27 HSV-1-infected HeLa cells (8 hpi). Data was scaled by row. (B) A scatter plot showing significant APA differences between mock- and ΔICP27 HSV-1-infected HeLa cells (8 hpi). (C) A Venn diagram showing the overlap between proximal PAS that displayed significant APA changes induced by the wild-type or ΔICP27 HSV-1. (D-F) PAS-seq tracks of mock-, wild-type HSV-1, or ΔICP27 HSV-1-infected HeLa cells at 8 hpi.

We recently showed that the HSV-1 immediate early factor ICP27 directly interacts with the mRNA 3’ processing factor CPSF and blocks mRNA 3’ end formation [13]. This suggests that ICP27 could be directly involved in HSV-1-mediated APA regulation. To test this possibility, we first compared the APA changes induced by the wild-type and the ΔICP27 HSV-1, in which the ICP27 gene was replaced by lacZ [24] in HeLa cells. The majority of HSV-1-induced APA changes were abolished or diminished in ΔICP27 infected cells (Fig 3A and S3A Fig, compare HSV1 and ΔICP27), strongly suggesting that ICP27 is required for HSV-1-mediated APA regulation. Interestingly, however, when comparing the APA profiles of mock and ΔICP27 infected cells, we detected 1,435 significant APA changes and the majority of these APA changes (1,109 genes or 77%) are DtoP shifts (Fig 3B). Therefore, the ΔICP27 virus induced an even greater number of APA changes than the wild-type virus. The APA changes induced by ΔICP27 virus seemed distinct from those by the wild-type virus. The proximal PAS involved in ΔICP27- and wild-type HSV-1-induced APA changes were largely distinct with relatively small overlap (310 genes in the overlap, Fig 3C). Additionally, 1,000 significant APA differences were detected when we compared the wild-type and ΔICP27 HSV-1-infected cells and 501 of these genes showed more proximal PAS usage in the wild-type virus-infected cells and 409 displayed the opposite trend (S3C Fig). These data demonstrate that HSV-1 can induce APA changes in both ICP27-dependent and -independent manners. For example, HSV-1 infection activated an intronic PAS in *EXOSC4* transcripts, and a similar activation was not observed in ΔICP27-infected cells (Fig 3D). By contrast, an intronic PAS in *CHTOP* is ICP27-independent as it was similarly activated in both wild-type and ΔICP27 HSV-1-infected cells (Fig 3E). Finally, an intronic PAS in *GLIS2* was only activated in ΔICP27- but not in wild-type HSV-1-infected cells (Fig 3F). Therefore, these results suggest that HSV-1 can induce APA changes through multiple mechanisms.

### Mechanisms for HSV-1-mediated APA regulation

**Fig 4:**
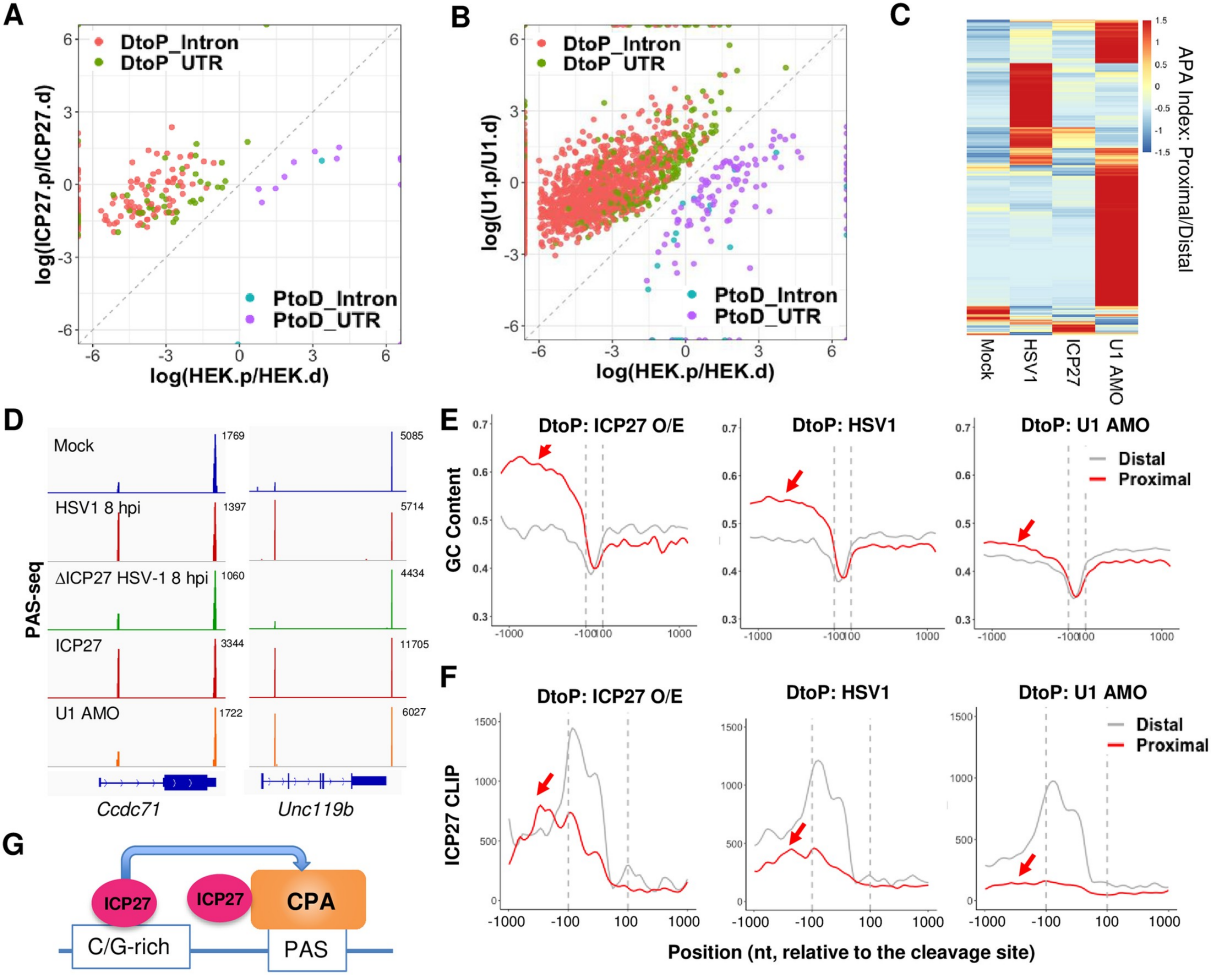
Mechanism of HSV-1-mediated APA regulation. (A) A scatter plot showing the significant APA changes induced by ICP27 over-expression in HEK293 cells. Color scheme and labeling are similar to Fig 1A. (B) A scatter plot showing the significant APA changes induced by U1 antisense morpholino oligo (AMO)-treatment of HEK293 cells. (C) A heat map of APA index of all genes that displayed significant APA changes induced by HSV-1-infection, ICP27 over-expression, or U1 AMO. Data was scaled by row. (D) PAS-seq tracks for two example genes. (E) GC content at the proximal and distal PAS of genes that displayed significant DtoP shifts induced by ICP27 over-expression (O/E), HSV-1 infection (HSV1), or U1 AMO treatment. (F) ICP27 CLIP signals at the proximal and distal PAS of genes that displayed significant DtoP shifts induced by ICP27 over-expression (O/E), HSV-1 infection (HSV1), or U1 AMO treatment.

Our data suggest that ICP27 is necessary for the majority of APA changes induced by wildtype HSV-1. We thus wanted to determine if ICP27 is sufficient to regulate APA. Based on RNA-seq analyses, the Krause laboratory recently provided evidence that ectopically expressed ICP27 regulates APA [25]. However, RNA-seq is not ideal for APA analysis as it lacks the sensitivity to detect APA changes of modest magnitude or those involving closely located alternative PAS [8]. To overcome these limitations, we performed PAS-seq analysis of mock-transfected or ICP27 over-expressing HEK293 cells. Overexpression of ICP27 induced significant APA changes in 169 genes, the vast majority of which (154 genes or 91%) were DtoP shifts (Fig 4A). Among these DtoP shifts, 111 genes or 72% shifted to a proximal intronic PAS (DtoP_Intron, Fig 4A). The majority of ICP27 overexpression-induced APA changes were also induced by HSV-1 infection (p < 5.1e-71, hypergeometric test; S4A Fig). However, the number of APA events regulated by ICP27 overexpression was significantly lower compared to that by HSV-1. Thus, although ICP27 is necessary for a majority of HSV1-induced APA changes, it is not sufficient to induce these changes.

Cleavage and polyadenylation at IPA are generally inhibited by the U1 snRNP [12]. As ICP27 overexpression primarily activates IPA, it was proposed that ICP27 may modulate APA by blocking U1 snRNP activity [25]. To test this model, we transfected U1 antisense morpholino oligo (AMO) into HEK293 cells, which blocks U1 snRNA-RNA interactions and thereby inhibiting U1 activity. PAS-seq analysis showed that U1 AMO treatment resulted in significant APA changes in 1,999 genes, the majority of which (1,867 genes or 93%) were DtoP shifts (Fig 4B). Consistent with previous studies, the majority of these APA changes (1,646 genes or 82% of the total) involve the activation of an intronic PAS (Fig 4B). A comparison between U1 AMO-, HSV-1- and ICP27-induced APA changes revealed largely distinct patterns with small overlaps (Fig 4C). For example, among the 169 genes whose APA is regulated by ICP27, only 32 (19%) are also regulated by U1 snRNP (S4B Fig). Similarly, 13% of HSV1-induced APA changes were also induced by U1 AMO (S4C Fig). For examples, ICP27 is necessary and sufficient to activate an IPA in *CCDC71* and *UNC119b* (Fig 4D). However, the intronic PAS of *UNC119b*, but not *CCDC71*, was induced by U1 AMO treatment (Fig 4D). These data suggest that HSV-1-mediated APA regulation does not involve a general inhibition of U1 snRNP.

To begin to understand the molecular basis for the specificity of HSV-1- and ICP27-induced activation of intronic PAS, we examined the sequences of the regulated PAS. Comparison of the IPA activated by either ICP27 or HSV-1 with the corresponding distal PAS in the 3’ UTR, revealed a higher GC content at the IPA (Fig 4E). ICP27-activated intronic proximal PAS are highly G/C-rich in the region upstream of cleavage sites (Fig 4E, left panel). HSV-1-activated intronic proximal PAS show an intermediate GC content (Fig 4E, middle panel). By contrast, intronic PAS activated by U1 suppression had a lower GC content (less than 50%, Fig 4E, right panel). We have previously shown that ICP27, when bound to upstream GC-rich sequences, can activate PAS (Fig 4G) [13]. To test if the GC contents of the different classes of IPA impact ICP27 binding, we took advantage of the ICP27 CLIP-seq dataset that we generated previously [13]. Interestingly, we commonly observed high ICP27 CLIP-seq signals upstream of ICP27-activated intronic PAS and intermediate levels of ICP27 CLIP-seq signals at HSV-1-induced IPA (Fig 4F, left and middle panels). By contrast, very little ICP27 binding was detected upstream of U1-regulated IPA (Fig 4F, right panel). Thus, the ICP27 CLIP-seq signal intensities at these IPA are highly consistent with the respective GC content. These observations are consistent with the model that ICP27 activates specific PAS by binding to GC-rich upstream sequences during HSV-1 infection, and that HSV-1-mediated APA regulation does not involve a general inhibition of U1 snRNP.

### Export of HSV-1-induced APA isoforms

**Fig 5:**
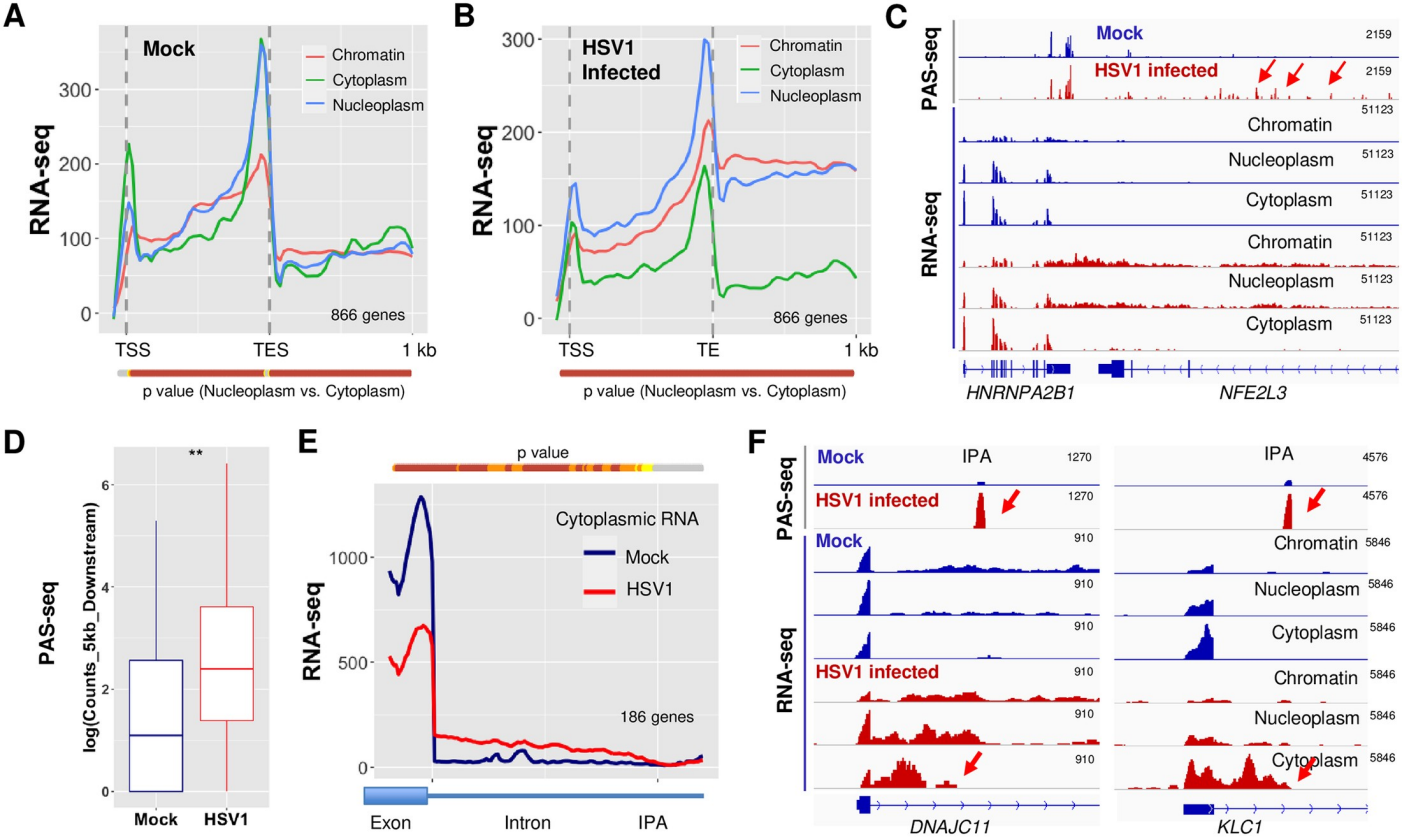
HSV-1-mediated APA regulation and mRNA export. Average RNA-seq signals for genes that display significant HSV-1-induced APA changes in chromatin, nucleoplasm, and cytoplasm fractions in mock-infected (A) or HSV-1-infected human fibroblast cells (B). P values for the difference in RNA-seq signals at each position between the nuclear and cytoplasmic fractions were calculated using Wilcoxon rank sum test and plotted as a color-coded bar below the plot. The color scheme is the same as Fig 2A. (C) PAS-seq and RNA-seq tracks for *HNRNPA2B1* gene. Red arrow points to the PAS-seq signals downstream of the normal TES. (D) PAS-seq signals in the 5kb region downstream of the normal TES in mock- and HSV-1-infected cells. **:P value < 0.05, Wilcox test. (E) Cytoplasmic RNA-seq signals for HSV-1-induced DtoP_intron APA changes. IPA: intronic poly(A) site. (F) PAS-seq and RNA-seq tracks for two example genes. Red arrows point to the IPA.

We next wanted to determine how HSV-1-induced APA changes regulate the export of the corresponding transcripts. To address this question, we analyzed a RNA-seq dataset that we have recently generated for chromatin, nucleoplasmic, and cytoplasmic fractions of mock- or HSV-1-infected human fibroblast cells at 8 hpi [17]. Our previous study showed that known nuclear lincRNAs, including MALAT1 and NEAT1, and cytoplasmic lincRNAs, including LINC00657 and VTRNA2-1, were enriched in nuclear and cytoplasmic fractions respectively [17]. In addition, intronic reads were over-represented in chromatin-associated fraction [17]. These observations suggested that the fractionation was efficient. To measure the overall export efficiencies of HSV-1 target APA isoforms, we performed a meta-analysis of all genes that displayed significant APA changes in HSV-1-infected cells, but do not overlap with other genes within the 1 kb region downstream of TES. Compared to the RNA-seq patterns in mock treated cells (Fig 5A), HSV-1-infected cells displayed two major differences (Fig 5B). First, there was significant accumulation of RNA-seq signals downstream of TES in the chromatin and nucleoplasm fractions, consistent with HSV-1-induced DoTT. Secondly, accumulation of RNAs was observed in the nucleoplasm relative to the cytoplasm in both gene body and downstream regions (Fig 5B), suggesting that indicating that the transcripts that extended past the TES were released into the nucleoplasm, but not exported. The release of DoTT transcripts could be due to cleavage/polyadenylation downstream of the normal TES. Indeed, as shown in Fig 5C, elevated levels of PAS-seq signals were observed downstream of *HNRNPA2B1* TES (PAS-seq peaks downstream of the TES are marked by red arrows). On the global level, we compared the PAS-seq reads in the 5 kilobase (kb) region downstream of the annotated TES for all PtoD genes and found that, indeed, there are significantly higher PAS-seq reads within this region in HSV-1-infected cells compared to mock treated cells (Fig 5D, p = 0.002, Wilcoxon test). The PAS-seq reads in the downstream regions contain the canonical poly(A) signal, AWTAAA hexamer, at -20 nt position (S5A Fig), and do not have a poly(A) run downstream of the cleavage sites (S5B Fig, strongly suggesting that these PAS-seq reads are due to the usage of cryptic PAS and not due to a potential technical artifact such as internal priming. These data suggest that HSV-1-induced extended transcripts as a result of DoTT are released into the nucleoplasm by cleavage/polyadenylation at cryptic PAS in the downstream region. However, these transcripts are not efficiently exported into the cytoplasm (Fig 5B and 5C).

HSV-1 infection activates IPA in a large number of genes (Fig 1A). The resultant transcripts are predicted to encode truncated proteins. To monitor the fate of these RNAs, we performed a meta-analysis of the region from the upstream exon to the intronic PAS for DtoP_intronic genes, which distinguishes the spliced and polyadenylated APA isoforms (Fig 5E). Signals from the upstream exon reflect both spliced and polyadenylated transcripts whereas the signals in the intronic region are only derived from the unspliced polyadenylated isoform. In the cytoplasm of mock infected cells, high RNA-seq signals were observed for the upstream exon while almost no signals were detected in the intronic regions (Fig 5E, blue line), suggesting that only fully spliced transcripts are exported. However, RNA-seq signals decreased in the upstream exon region, but accumulated between the upstream exon and the IPA in the cytoplasm of HSV-1-infected cells (Fig 5E, red line). This suggests that the transcripts polyadenylated at IPA are exported into the cytoplasm. Two examples were provided in Fig 5F. For both *DNAJC11* and *KLC1*, their transcripts are efficiently spliced in mock treated cells, but HSV-1 infection activates a PAS within the first intron, as shown by the PAS-seq data (Fig 5F, PAS-seq tracks, activated IPA are marked by red arrows). Our fractionation RNA-seq data showed that these truncated RNA isoforms are exported into the cytoplasm (Fig 5F, RNA-seq tracks, cytoplasmic tracks are marked by red arrows). These results are highly consistent between the two biological replicates of sub-cellular fractionation in our dataset (S5C Fig). Based on these observations, we conclude that the transcripts of the APA target genes are exported less efficiently and that the truncated transcripts polyadenylated at intronic PAS are exported. We further estimated the IPA isoform export efficiency by calculating the ratio of RNA-seq signals within the 500 nt region upstream of the intronic PAS to the 500 nt region downstream in all APA changes involving proximal IPA (S5D and S5E Fig). The results suggest that the intronic reads upstream of the IPA increased for DtoP genes (S5C Fig), but decreased in PtoD genes (S5D Fig), further suggesting that the IPA isoforms are exported.

### Translation of HSV-1-induced APA isoforms

**Fig 6:**
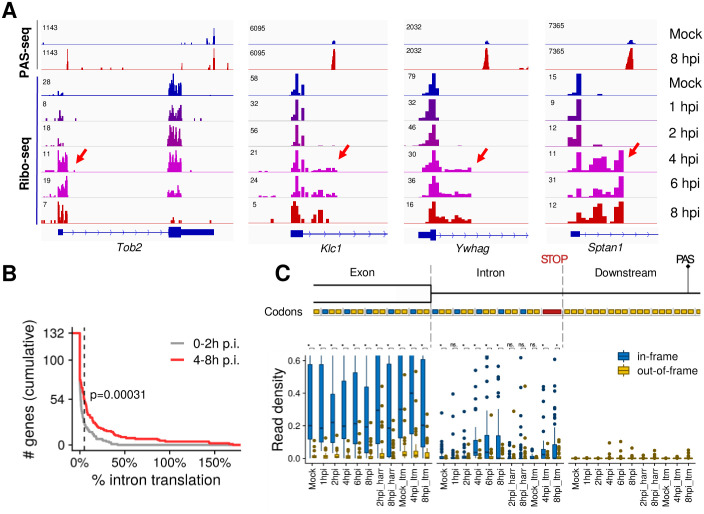
HSV-1-induced intronically polyadenylated APA isoforms are translated. (A) PAS-seq and Ribo-seq tracks for four example genes. Ribo-seq signals within intronic regions are marked by red arrows. (B) Cumulative distribution of the percentage of intronic Ribo-seq read densities (normalized reads per codon) compared to the level in the upstream exon. The dashed line indicates genes exceeding the 5% threshold mentioned in the text. The P value for comparing the pooled read densities in un-infected through 2 hpi vs. 4–8 hpi is shown (two-sided Kolmogorov-Smirnov test) (C) Boxplots showing the distributions of read densities for the 54 genes exceeding the 5% threshold stratified by time point after infection, location with respect to exon-intron boundary and first intronic stop codon, and reading frame of translation. The hinges and whiskers correspond to quartiles and to the most extreme values outside of 1.5 times the inter-quartile range, respectively. The median and outliers are indicated. The y axis is arbitrarily cut at 0.6. P values for comparisons of in-frame and out-of-frame codons are indicated (***, p<0.001; **, p<0.01; *, p<0.05; n.s., not significant at 5% level).

Our data suggests that at least a subset of the HSV-1-induced APA isoforms polyadenylated at IPA are exported into the cytoplasm (Fig 5E and 5F and S5C and S5D Fig), raising the question whether they are translated. To test this, we examined our Ribo-seq dataset for HSV-1-infected human fibroblast cells at different time points post-infection. In un-infected cells, the Ribo-seq signals were limited to exonic regions as expected (Fig 6A). Interestingly, however, Ribo-seq signals extended into intronic regions within many of the HSV-1-induced APA isoforms (Fig 6A). These results not only further support our conclusion that intronically polyadenylated APA isoforms can be exported into the cytoplasm, but also indicate that they are engaged with ribosomes. For 54 out of 132 intronically polyadenylated APA isoforms that had least 5 intronic codons upstream of the first intronic stop codon and where the upstream exon was translated, the intronic read density exceeded 5% of the upstream exon at 4–8 hpi. Several lines of evidence indicate that these reads correspond to ribosomes that continue translation elongation from the upstream exon into the intron: 1) Intronic translation was much weaker in un-infected samples and during early infection (before 4 hpi) (Fig 6B; p = 3.1x10^-4^, Kolmogorov-Smirnov test); 2) Mapping Ribo-seq reads to nucleotide resolved ribosome positions [26] revealed a strong enrichment of in-frame codons, providing strong evidence for actively translating ribosomes (Fig 6C); 3) Virtually no reads were observed downstream of the first intronic in-frame stop codon (Fig 6C); 4) Lactimidomycin (ltm) or harringtonine (harr) treated samples, in which translation is stalled at the initiation stage [26], exhibited lower intronic read densities, suggesting that the Ribo-seq signal is not due to spurious intronic translation initiation (Fig 6C and S6 Fig). Taken together, these results provide strong evidence that at least some of the HSV-1-induced IPA isoforms are translated at considerable levels.

## Discussion

**Fig 7:**
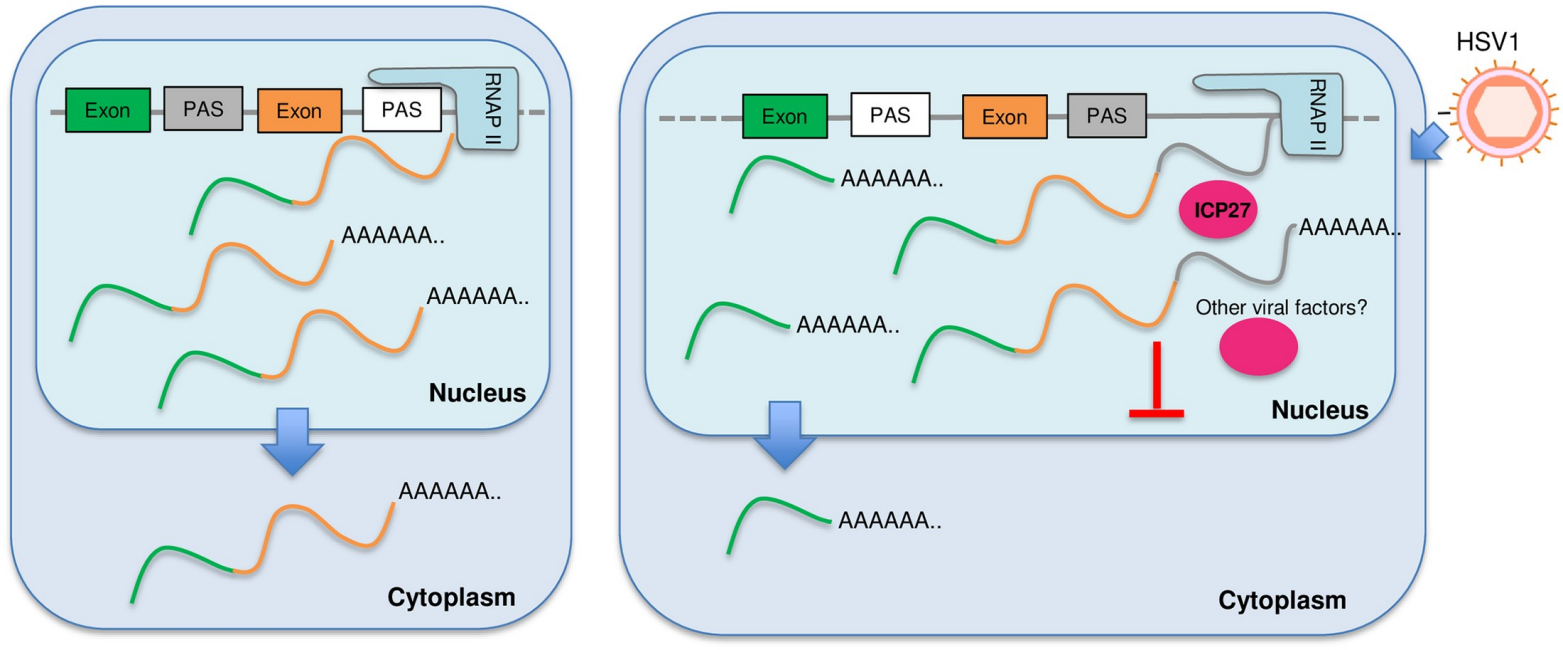
A model for HSV-1-mediated APA regulation. In HSV-1-infected cells, ICP27 and other viral factors induce many APA changes and transcription termination defects. Transcripts that extend beyond the normal TES are cleaved and polyadenylated, but are not exported. Truncated transcripts that are polyadenylated at IPA can be exported into the cytoplasm and translated. Please see text for more details.

mRNA 3’ end processing and transcription termination are tightly coupled processes. Viral infections (HSV-1 and IAV) and cellular stresses (salt/osmotic stress and heat shock) induce DoTT/DoG [14–16]. Meanwhile, several pathogens, including viruses (HCMV and VSV) and bacteria (listeria and salmonella), as well as arsenic stress causes widespread APA changes [18–20,27]. However, no study has characterized these two processes in response to the same pathogen or stress. In this report, we performed extensive transcriptomic analyses of wild-type and mutant HSV-1-infected cells and found that lytic HSV-1 infection induced widespread APA changes in host transcripts, the majority of which shifted to upstream PAS. HSV-1-mediated APA regulation requires the viral immediate early factor ICP27 as well as other viral factors, but does not involve a general inhibition of U1 snRNP. Interestingly, HSV-1 induces both activation of upstream PAS with pre-mature transcription termination and a termination defect. Activation of upstream intronic PAS produces truncated transcripts that are exported into the cytoplasm and translated. By contrast, although extended transcripts due to DoTT can be cleaved and polyadenylated at downstream cryptic PAS, these transcripts are sequestered in the nucleoplasm. Together, these results demonstrate that HSV-1-mediated regulation of APA and transcription termination profoundly reprograms host transcriptomes (Fig 7).

Although widespread APA changes have been described for a number of pathogen-infected cells and for cells exposed to arsenic stress [18–20,27], the underlying mechanism remains poorly understood. Our data suggest that HSV-1 induces APA changes of host mRNAs through multiple mechanisms. First, the viral immediate early factor ICP27 contributes to HSV-1-induced APA changes. We have previously shown that ICP27 has bimodal activities: it broadly inhibits mRNA 3’ processing through direct interactions with the 3’ processing factor CPSF, but can activate PAS that contain GC-rich upstream sequences [13]. Indeed, both HSV-1 infection and over-expression of ICP27 can activate upstream intronic PAS and these PAS contains GC-rich upstream sequences (Fig 4E and 4F). 3’ processing at these intronic PAS induces early termination (Fig 2A–2C). On the other hand, the corresponding downstream PAS in these genes lack GC-rich upstream sequences and are thus inhibited, leading to DoTT at these sites. Thus, the bimodal activities of ICP27 provide an explanation for the paradoxical observation of early termination and DoTT in these genes. Secondly, our observation that the ΔICP27 virus still induce a large number of APA. Similarly, we have previously shown that ΔICP27 HSV-1 also induced DoTT, albeit at lower levels compared to that by the wild-type virus [13]. These results suggest that other mechanisms are also involved. Although ICP27 is required for viral replication and for the expression of early and late genes, the ΔICP27 virus still contains the tegument proteins VP16 and vhs, and other immediate early proteins, such as ICP4, ICP0 and ICP22 are also expressed [28]. Thus the viral DNA and other viral proteins may induce APA changes and DoTT either directly through interactions with host mRNA 3’ processing factors or indirectly. Since multiple pathogens and stress induce similar changes in both APA and DoTT, it is likely that a common mechanism underlies these phenomena. One possibility is that viral infections and cellular stress may alter the activity of RNAPII. In addition to its role in transcribing genes, RNAPII also plays an essential role in coordinating transcription and RNA processing primarily through its C-terminal domain. Both phosphorylation and dephosphorylation of RNAPII CTD have been shown to influence termination [4–6,29]. For example, pharmacological or genetic inhibition of Cdk12, which phosphorylates RNAPII CTD at serine 2, leads to activation of intronic PAS and premature termination [30,31]. On the other hand, PP1 or PP2A, phosphatases that dephosphorylate RNAPII CTD, play essential roles in regulating transcription pausing and termination [5,32–34]. Previous studies provided evidence that HSV-1 infection induces aberrant CTD phosphorylation and partial degradation of RNAPII [35,36]. It will be important to characterize RNAPII post-translational modifications and interactomes in pathogen-infected and in stressed cells and determine if/how such changes contribute to the virus-induced APA changes and DoTT.

The functional consequence of pathogen/stress-induced DoTT and APA changes remains unclear. The most important functions of stress responses are to: 1) shut down the expression of most genes to avoid accumulation of aberrant proteins; 2) activate stress response genes to stabilize and repair biomolecules [37]. Similarly, when a pathogen infects a host cell, it shuts down host gene expression and hijacks the host machinery to express genes of the pathogen. Both DoTT and APA changes could contribute to the repression of cellular genes. DoTT interferes with the transcription cycle and prevents mRNA biogenesis. Consistent with previous reports [17], our results showed that at least some of the read-through transcripts as a result of DoTT are in fact cleaved and polyadenylated, and released into the nucleoplasm (Fig 5). However, they are not efficiently exported. On the other hand, HSV-1-induced activation of upstream intronic PAS leads to the production of truncated transcripts that can be exported (Fig 5E). We provide evidence that at least some these truncated mRNAs are translated (Fig 6). Therefore, both DoTT and APA may function in host shutoff (for pathogens) or repressing bulk gene expression (for stresses). Alternatively, the DoTT and APA changes observed in pathogen-infected or stressed cells could represent a host defense mechanism. Previous studies provided evidence that arsenic stress-induced APA isoforms with shorter 3’ UTRs, which can evade RNA degradation, are thus better preserved [20]. This may facilitate better recovery from stress. VSV-induced APA changes have been shown to modulate the innate immunity response [19]. In summary, pathogen- and stress-induced APA changes may function in host shut-off or in host defense, and these two mechanisms are not mutually exclusive.

## Methods

### Cell culture, viruses and infection

HEK293 and HeLa cell lines were cultured in Dulbecco’s modified Eagle medium (DMEM) with 10% fetal bovine serum (FBS). All cells were incubated at 37°C in a 5% (v/v) CO2-enriched incubator. Virus stocks for wild-type HSV-1 strain KOS as well as the ICP27 null mutant (strain KOS) [24] were produced on complementing Vero 2–2 cells [38]. HeLa cells were infected with an MOI of 10 unless otherwise specified and incubated at 37°C until cells were harvested at the specified time points. For anti-sense morpholino oligo treatment, HEK293 cells were treated with 50 μM U1 antisense morpholino oligo (AMO) (Gene tools) and 10 μM Endo-Porter (Gene tools). After 48 hours, RNA was extracted by using Trizol (Ambion). ICP27 over-expression was also performed in HEK293 cells.

### PAS-seq

Total RNA was extracted with Trizol as per manual (Life technologies), 10 μg total RNA was fragmented with fragmentation reagent (Ambion) at 70°C for 10 minutes followed by precipitation with ethanol. After centrifugation, RNA was dissolved and Reverse transcription was performed with PASSEQ7-2 RT oligo: [phos]NNNNAGATCGGAAGAGCGTCGTGTTCGGATCCATTAGGATCCGAGACGTGTGCTCTTCCGATCTTTTTTTTTTTTTTTTTTTT[V-Q] and Superscript III. cDNA was recovered by ethanol precipitation and centrifugation. 120–200 nucleotides of cDNA was gel-purified and eluted from 8% Urea-PAGE. Recovered cDNA was circularized with Circligase^™^ II (Epicentre) at 60°C overnight. Buffer E (Promega) was added in cDNA and heated at 95°C for 2 minutes, and then cool to 37°C slowly. Circularized cDNA was linearized by adding BamH I (Promega). cDNA was centrifugated after ethanol precipitation. PCR was carried out with primers PE1.0 and PE2.0 containing index. Around 200 base pairs of PCR products was gel-purified and submitted for sequencing (single read 100 nucleotides). PAS-seq samples include: HSV-1-infected HeLa cells at 0, 2, 6, and 12 hpi (one for each time point); mock-, wild-type HSV-1, or ΔICP27 HSV-1-infected HeLa cells at 8 hpi (one for each); mock-, ICP27 over-expressing, and U1 AMO-treated HEK293 cells (one for each). For APA analysis, mock-treated and HSV-1-infected HeLa cells at 0 hpi were considered as biological replicates and HSV-1-infected HeLa cells at 6 and 8 hpi as replicates.

### PAS-Seq data analysis

From the raw PAS-seq reads, first those with no poly(A) tail (less than 15 consecutive “A”s) were filtered out. The rest were trimmed and mapped to hg19 genome using STAR. If 6 consecutive “A”s or more than 7 “A”s were observed in the 10 nucleotide downstream of PAS for a reported alignment, it was marked as a possible internal priming event and removed. The bigwig files were then generated for the remaining reads using deepTools (v2.4) with “normalizeUsingRPKM” and “ignoreDuplicates” parameters [39].

Next, the locations of 3’ ends of the aligned reads were extracted and those in 40nt of each other were merged into one to provide a list of potential PAS for human. This list was then annotated based on the canonical transcripts for known genes. The final read count table was created using the reads with their 3’ ends in -40nt to 40nt of these potential PAS.

Alternatively polyadenylated PAS in different experimental conditions were identified using diffSpliceDGE and topSpliceDGE from edgeR package(v3.8.5) [40]. This pipeline first models the PAS read counts for all PAS, then compares the log fold change of each PAS to the log fold change of the entire gene. This way, these functions, primarily used to find differential exon usage, generate a list of sites with significant difference between our PAS-seq samples. From this list, those with a FDR value less than 0.05 and more than 15% difference in the ratio of PAS read counts to gene read counts (normalized by sequencing depth) between samples were kept, and finally for each gene the top two were chosen based on P-value and marked distal or proximal based on their relative location on the gene. For PAS-seq comparisons without replicates, Fisher’s exact test was used to compare read counts at a PAS and the total read counts from the same gene. The P values were adjusted by the Benjamini–Hochberg method for calculating the FDR.

For the genes with alternatively polyadenylated sites (target genes), the log2 of ratio of read counts in the distal site to the read counts in the proximal site was calculated and illustrated as a heatmap in Figs 1C, 2A and 3C with pheatmap in R. The heatmap is hierarchically clustered using Pearson correlation of the gene profiles in different experiments.

### Ribo-seq analysis

We applied Bowtie 1.0 (REF) to map reads to rRNA, genomic and transcriptomic sequences from the Ensembl database (version 75). rRNA reads and reads mapping to the mitochondrial genome were discarded. All alignments were mapped to genomic coordinates. Fractional counts were used for ambiguous alignments (with regard to genomic coordinates). We then used the probabilistic model implemented in Price (version 1.0.3b) [26] to map reads to their P site codons using default parameters. All read counts corresponding to translation start site profiling (lactimidomycin-treated samples) were discarded. Next, we removed (i) PAS that were located inside of Ensembl 75 exons (n = 110), (ii) PAS without an annotated or Price-identified open reading frame (ORF) in the upstream exon (n = 64), (iii) PAS without an in-frame stop codon in the intron in between the exon boundary and the PAS (n = 9), (iv) PAS where the first in-frame stop codon was in the first five intronic codon triplets (n = 100), and (v) PAS downstream of very weakly translated ORFs (<0.5 reads per exonic codon in all Ribo-seq samples pooled; n = 104). For the remaining n = 132 PAS, we counted codon mapped reads for the partial open reading frame in the upstream exon (reads mapped to codons in the same frame as the ORF, and in the other two frames), for its extension into the intron up to the first in-frame stop codon (reads mapped to codons in the same frame as the ORF, and in the other two frames), and reads mapped in between the stop codon and the PAS (Fig 6C). Read counts were normalized to the total number of Ribo-seq reads mapped to the human genome.

### Meta-analysis

Meta-analyses of read distribution were performed using deeptools [39]. 4SU-seq, iCLIP-seq, or RNA-seq reads were first mapped to the human genome (hg19), and then normalized by library size to produce bigwig files using the bamCoverage tool in deepTools. Variable sized regions (gene body or the region between TSS and IPA) were divided into 100 bins. Fixed sized regions were divided into 10 nt bins. Sequencing signal scores for each bin were calculated using deepTools. For meta-analyses in Fig 2, signal scores for each gene were further normalized by their sum before calculating the average scores. To evaluate the statistical significance of the meta-analysis results in Figs 2 and 5, p values were calculated for the sequencing signal scores of all the genes in mock and HSV-1 samples at each nucleotide position using the Wilcoxon rank sum test, and the results are showed as color-coded bars under each plot. For the analyses shown in Figs 2 and 5A and 5B, additional filtering was performed to remove the genes that overlap with other genes within the 1kb downstream region. For the analysis in Fig 5E, DtoP genes were filtered to keep only those whose first intronic PAS was activated by HSV-1 infection to minimize the influence of different annotations of upstream exons.

### Data and software availability

RNA-seq data on the subcellular RNA fractions, 4sU-seq, and Ribo-seq data were previously published [13,17]. PAS-seq data have been deposited to the GEO database (GSE151104).

## Supporting information

S1 Fig (A) Normalized PAS-seq read counts at proximal and distal PAS of APA genes. (B) A heat map of log(APA index) scaled by column.
(TIFF)

S2 Fig A boxplot of 4sU-seq signal ratio between the regions 500nt upstream and 500nt downstream of the intronic PAS of DtoP genes. (TIFF)

S3 Fig (A) A heat map showing the log(APA index) for genes with significant APA changes between mock and HSV-1-infected cells (scaled by column). (B) A heatmap showing the APA index (Proximal/Distal read counts) for all genes that displayed significant APA changes in either KOS or dICP27-infected cells (combination of genes in Fig 2A and 2B). Data scaled by row. (C) A scatter plot showing the significant APA changes between wild-type (KOS) and ΔICP27 (dICP27) virus-infected cells.
(TIFF)

S4 Fig Venn diagrams showing comparison of significant APA changes in U1 AMO-treated and ICP27 over-expressing or KOS-infected cells. P values were calculated using the hypergeometric test.
(TIFF)

S5 Fig (A) Distribution of AWTAAA motif at the PAS-seq peaks downstream of normal TES. (B) Nucleotide composition of cryptic PAS downstream of normal PAS. (C) RNA-seq tracks of cytoplasmic RNAs in mock- and HSV-1-infected cells (8 hpi). The ratio of RNA-seq read counts in the 500 nt upstream and 500 nt downstream of the intronic PAS for DtoP (D) and PtoD (E) genes for the cytoplasmic fractions of mock- and HSV-1-infected cells.
(TIFF)

S6 Fig Ribo-seq tracks for example genes (related to Fig 6A). Lactimidomycin is a translation inhibitor.
(TIFF)
