# Supplementary figures and images for: Mechanism and consequences of herpes simplex virus 1-mediated regulation of host mRNA alternative polyadenylation
Source: PLoS Genet. 2021 Mar 8;17(3):e1009263. doi: 10.1371/journal.pgen.1009263 (PMC7971895; doi:10.1371/journal.pgen.1009263)

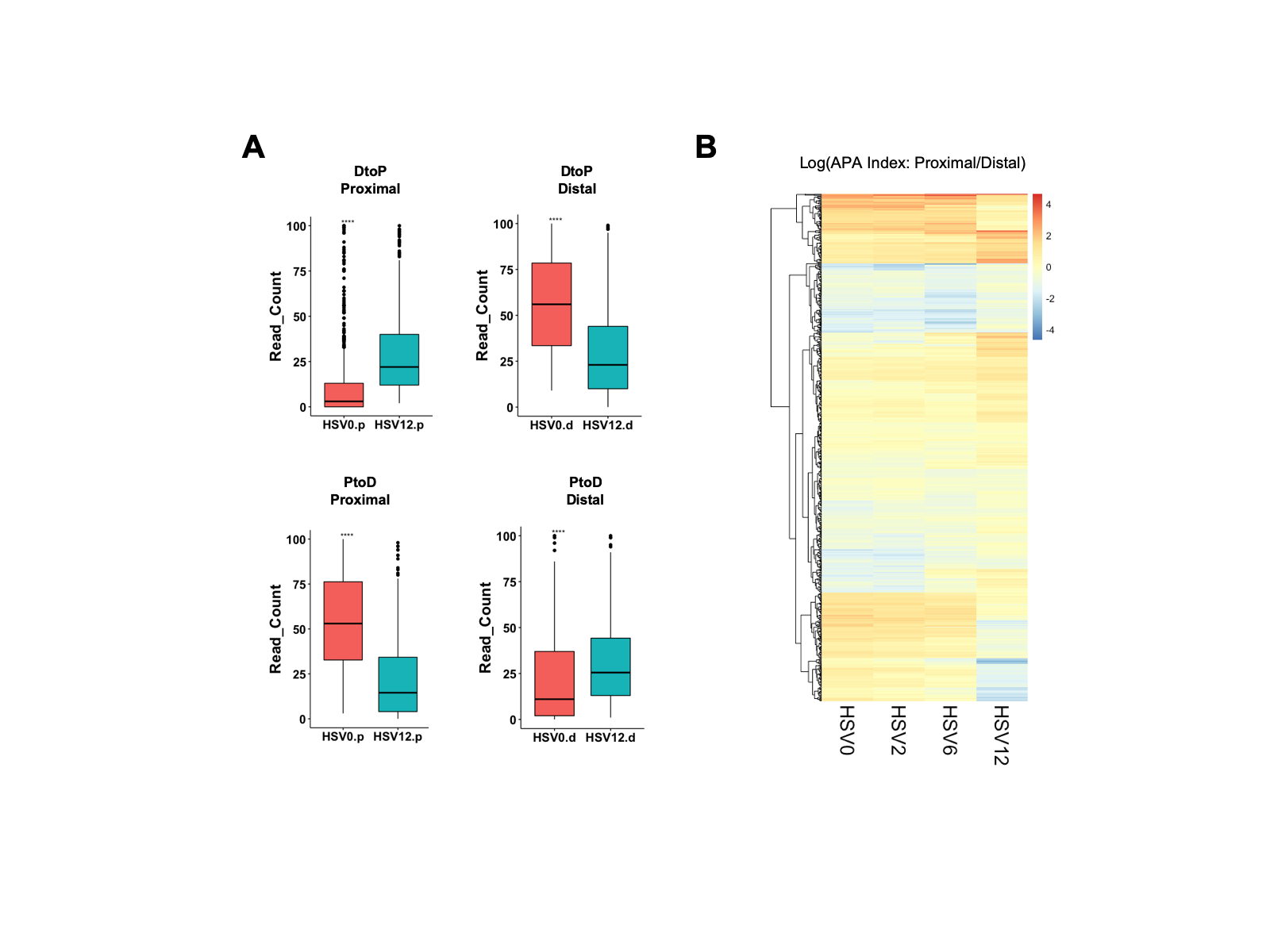

Supplement: S1 Fig — (A) Normalized PAS-seq read counts at proximal and distal PAS of APA genes. (B) A heat map of log(APA index) scaled by column. (TIFF) [file pgen.1009263.s001.tiff]

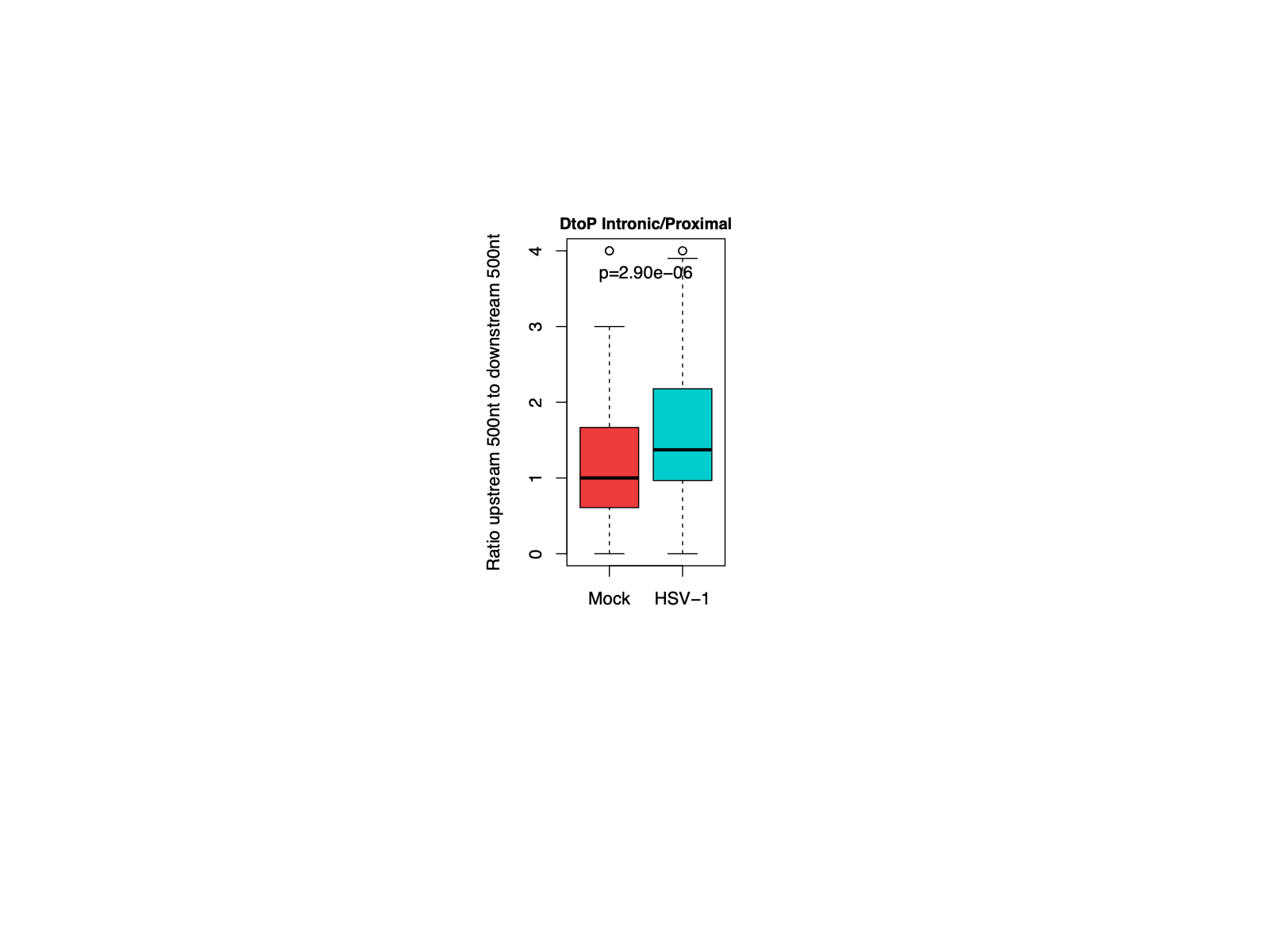

Supplement: S2 Fig — (TIFF) [file pgen.1009263.s002.tiff]

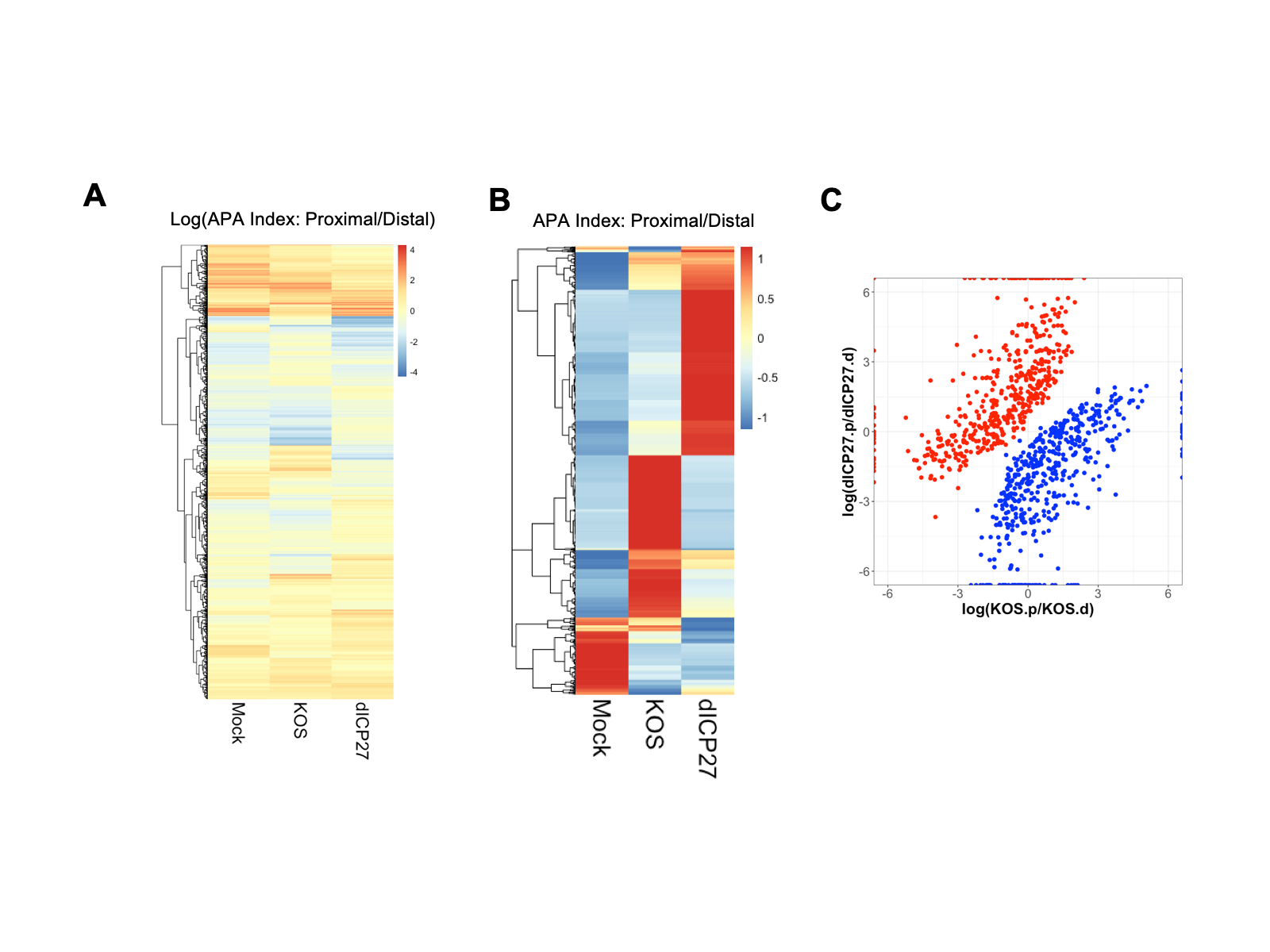

Supplement: S3 Fig — (A) A heat map showing the log(APA index) for genes with significant APA changes between mock and HSV-1-infected cells (scaled by column). (B) A heatmap showing the APA index (Proximal/Distal read counts) for all genes that displayed significant APA changes in either KOS or dICP27-infected cells (combination of genes in Fig 2A and 2B). Data scaled by row. (C) A scatter plot showing the significant APA changes between wild-type (KOS) and ΔICP27 (dICP27) virus-infected cells. (TIFF) [file pgen.1009263.s003.tiff]

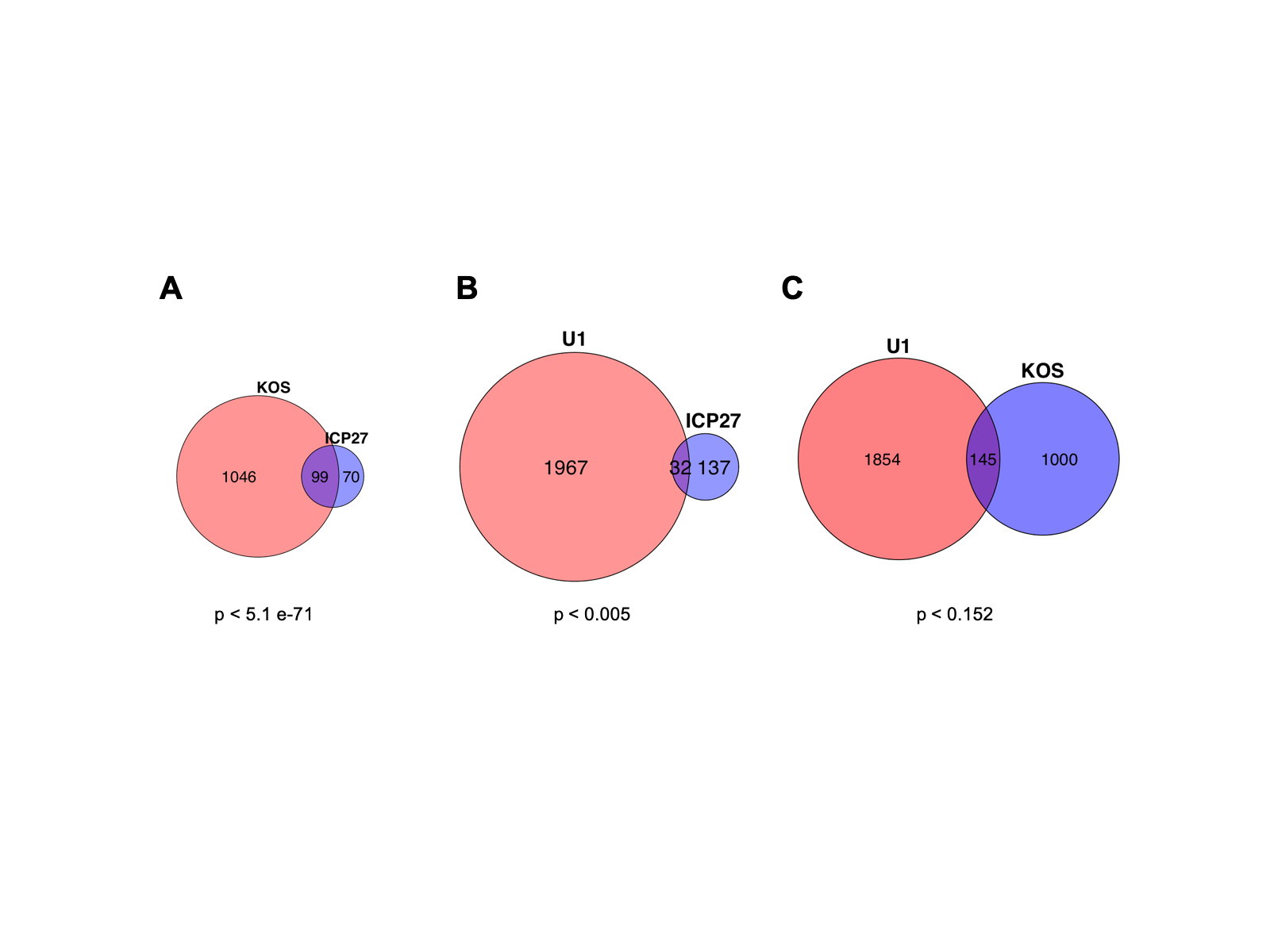

Supplement: S4 Fig — P values were calculated using the hypergeometric test. (TIFF) [file pgen.1009263.s004.tiff]

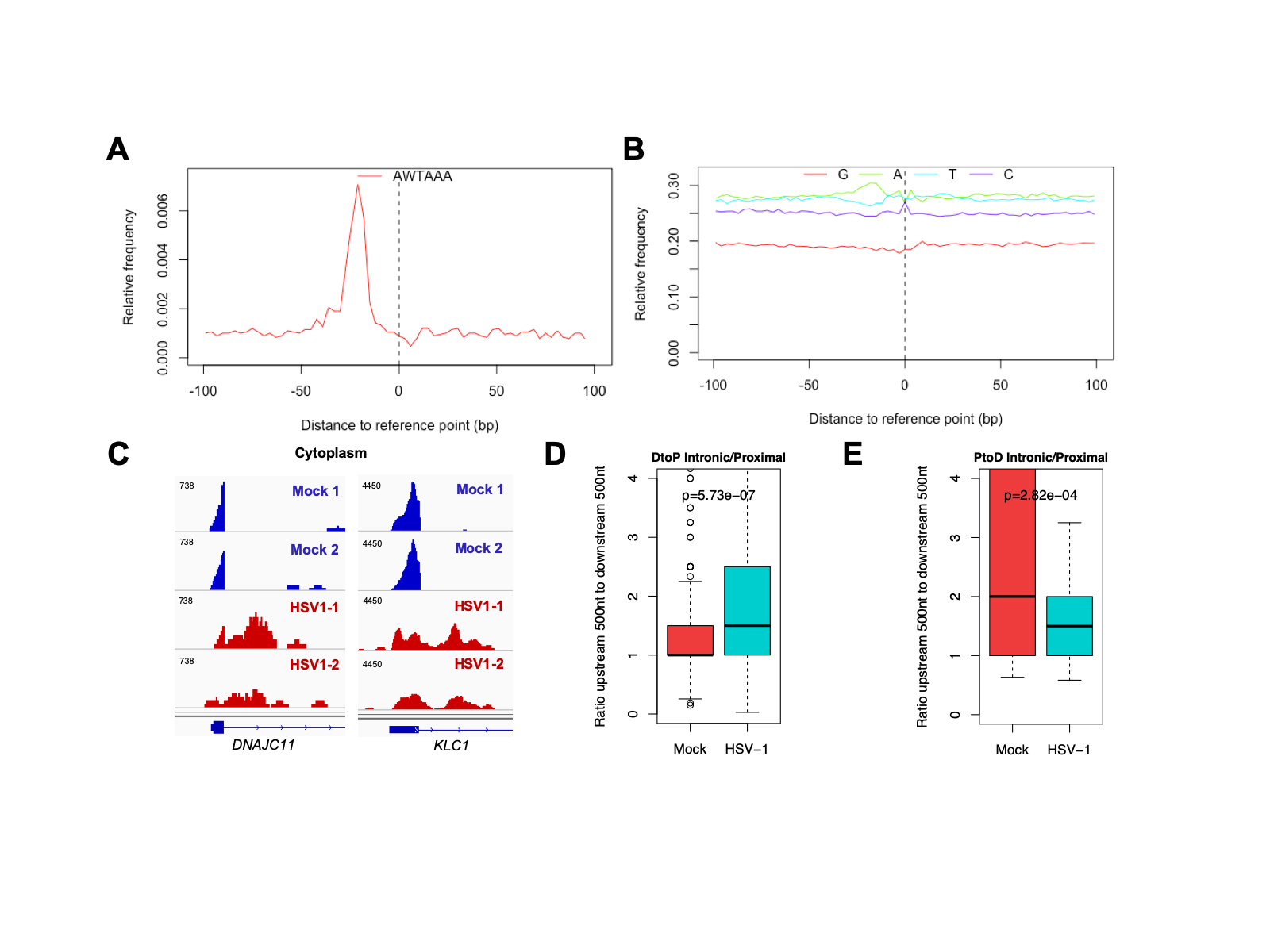

Supplement: S5 Fig — (A) Distribution of AWTAAA motif at the PAS-seq peaks downstream of normal TES. (B) Nucleotide composition of cryptic PAS downstream of normal PAS. (C) RNA-seq tracks of cytoplasmic RNAs in mock- and HSV-1-infected cells (8 hpi). The ratio of RNA-seq read counts in the 500 nt upstream and 500 nt downstream of the intronic PAS for DtoP (D) and PtoD (E) genes for the cytoplasmic fractions of mock- and HSV-1-infected cells. (TIFF) [file pgen.1009263.s005.tiff]

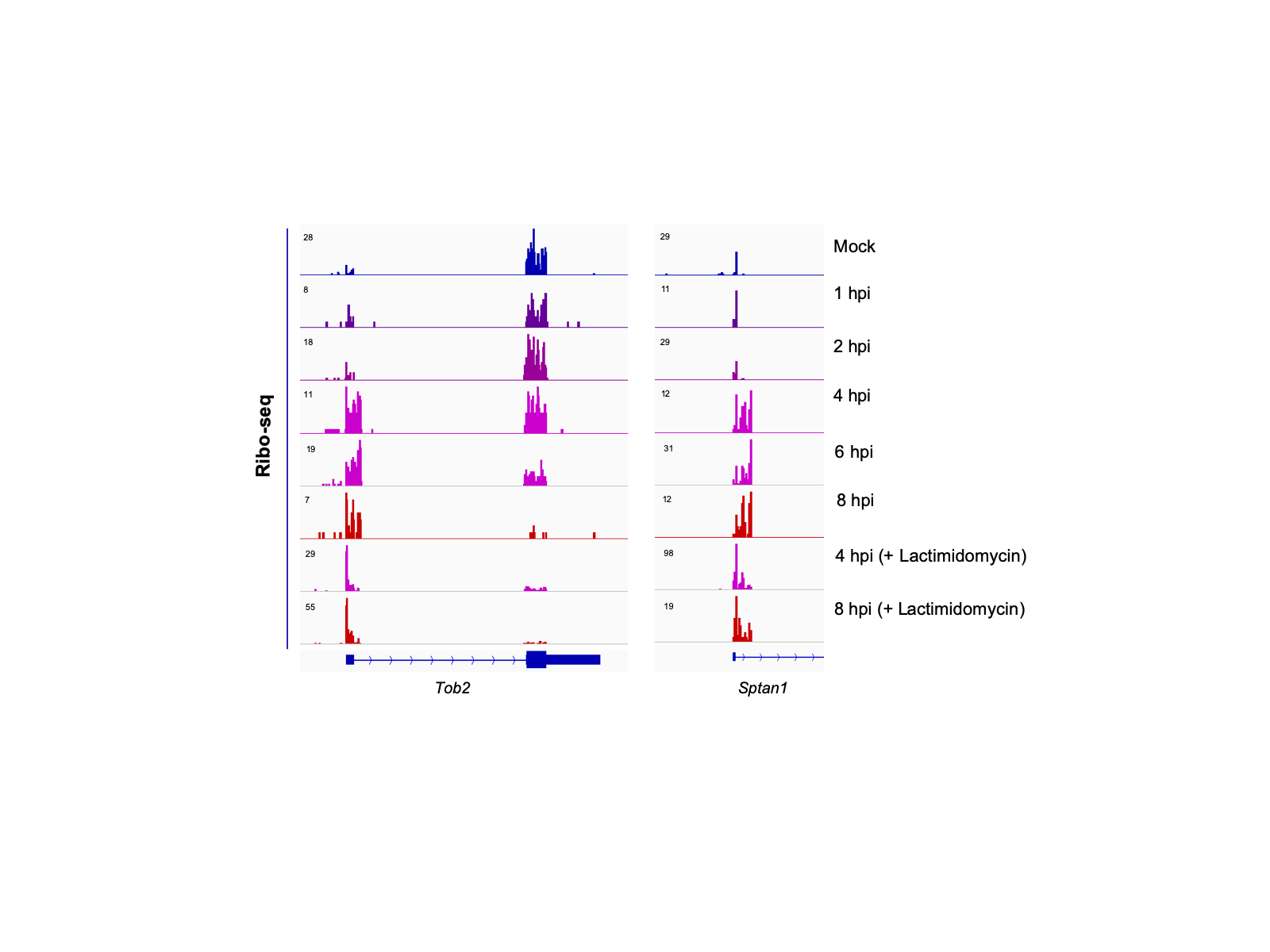

Supplement: S6 Fig — Lactimidomycin is a translation inhibitor. (TIFF) [file pgen.1009263.s006.tiff]
